# Special Issue: “Optimising Nutrition to Alleviate Age-Associated Functional Decline”

**DOI:** 10.3390/nu13082824

**Published:** 2021-08-17

**Authors:** Tomasz Kostka

**Affiliations:** Department of Geriatrics, Healthy Ageing Research Centre, Medical University of Lodz, Plac Hallera 1, 90-647 Lodz, Poland; tomasz.kostka@umed.lodz.pl

Appropriate nutrition is a cornerstone of preventive gerontology. This Special Issue of *Nutrients* provides new insights on nutritional assessment and potential modifications of nutritional behaviours and supplements to prevent age-associated disorders and increase life expectancy in different populations of older subjects.

This Special Issue includes five original articles and four systematic reviews. The first review discusses the synergistic action of selected antioxidant micronutrients (vitamin C, vitamin E, selenium, and zinc) for inhibiting oxidative stress and DNA damage. Micronutrients are involved in every cellular/biochemical process. Seniors are prone to micronutrient deficiencies due to age-associated physiological changes and often poor diet. Moreover, the lack of micronutrients has an indirect impact on the genome. Their low levels reduce the activity of antioxidant enzymes and therefore inhibit the efficiency of defense against free radicals, which can lead to the formation of DNA lesions. The more DNA damage in the genetic material, the faster aging at the cellular level and the higher the risk of pathological processes (e.g., carcinogenesis). Targeted supplementation of crucial antioxidative micronutrients such as selenium, zinc, vitamin C, and vitamin E seems to have the potential to positively influence the condition of an ageing organism, including minimizing inflammation, enhancing antioxidative defense, and limiting the formation of DNA lesions. Consequently, it may lead to lowering the risk and incidence of age-related diseases such as cardiovascular diseases, neurodegenerative diseases, and malnutrition [1].

The continuous increase in life expectancy results in a growing risk of cancer, which consequently increases the population of older adults with cancer. In the second review, problems associated with diet and nutrition in the elderly undergoing active cancer therapy have been presented. As epigenetics, an emerging element of the regulation of gene expression, is involved in both aging and cancer and the epigenetic profile can be modulated by the diet, it seems to be a candidate to assist with planning a nutritional intervention in elderly populations with cancer. Nutritional interventions modulating the epigenetic profile, including caloric restriction and basal diet with modifications (elimination diet, supplementary diet), are discussed as the ways to improve the efficacy of cancer therapy and maintain the quality of life of older adults with cancer [2].

Nutritional interventions have been shown to be especially effective for cardiometabolic risk. The Mediterranean diet, with olive oil as a vital component, has both health benefits and acceptable adherence. The third review provides an updated overview of current knowledge on the benefits of olive oil most relevant to menopause-associated metabolic syndrome, including an analysis of the components with the greatest health impact, their effect on basic mechanisms of disease, and the state of the art regarding their action on the main features of metabolic syndrome [3].

Neurological diseases have steadily increasing significance for the health of aging populations. Citicoline is a chemical compound involved in the synthesis of cell membranes with a promising role in neurology. Citicoline is often used to enhance cognitive functions. In the fourth review, accessible databases were searched for articles regarding citicoline use in neurological diseases. The review found that citicoline has been proven to enhance cognitive functions among healthy individuals and improve prognosis after stroke. In an animal model of nerve damage and neuropathy, citicoline stimulated regeneration and lessened pain. Citicoline has a wide range of effects and could be an essential substance in the treatment of many neurological diseases [4].

Depression is one of the diseases with increasing prevalence in the older population. The first original article analyzes the relationship between nutritional status and depression symptoms severity in 1975 older outpatients. Women with higher-severity depression symptoms had significantly lower nutritional status, shorter education time, smaller calf circumference, and higher waist to height ratio. Men with depression symptoms had lower nutritional status, shorter education, and smaller calf circumference. In the model of stepwise multiple regression, nutritional status and education years were the only independent variables predicting the severity of depression symptoms in both women and men. Results obtained in the study indicate a strong relationship between proper nutritional status and education level with severity of depression symptoms in older women and men [5].

The second original article analyses the role of serotonin and other tryptophan (TRP) metabolites generated in the kynurenine pathway in the pathogenesis of depression. Ninety subjects in three groups, 30 subjects each, were enrolled in this study: controls (healthy young adults, group I) and older individuals without (group II) or with (group III) symptoms of mild and moderate depression. The average daily intake of TRP was significantly lower in group III than the remaining two groups, but group III was also characterised by higher urinary levels of L-kynurenine, kynurenic acid, xanthurenic acid, and quinolinic acid compared with younger adult individuals and older patients without mood disorders. Therefore, mild and moderate depression in the elderly may be associated with a lower intake of TRP and changes in its kynurenine metabolic pathway, which suggests a potential dietary TRP-based intervention in this group of patients [6].

The last three original articles assess nutritional status in hospitalised older adults. The first one presents an optimal set of variables that are independently associated with the mortality risk of 433 older comorbid adults that have been discharged from the geriatric ward. Stepwise backward variable selection and the iterative Bayesian model averaging approaches to the Cox proportional hazards models were used. The results of the multivariable analysis identified seven explanatory variables that were independently associated with the length of survival. The mortality rate was higher in males than in females; it increased with the comorbidity level and C-reactive proteins plasma level but was negatively affected by a person’s mobility, geriatric nutritional risk index (GNRI), and lymphocyte count, as well as the vitamin D plasma level [7].

The second study compares two widely recommended short nutrition assessment tools—Nutrition Risk Screening 2002 (NRS-2002) and Subjective Global Assessment Form (SGA)—with other Comprehensive Geriatric Assessment (CGA) measurements in 622 consecutively hospitalised older subjects. Both NRS-2002 and SGA were inversely related to anthropometric measurements, functional assessment tests, and Mini-Mental State Examination (MMSE) and positively associated with the Vulnerable Elders Survey-13 (VES-13) score. Comparison of well-nourished subjects and patients with suggested problems with nutrition according to NRS-2002 (0–2 vs. 3–7) and SGA (A vs. B + C) gave comparable results. Both nutritional scales at given cut-off points similarly discriminated anthropometric data and other CGA tools in the populations of well-nourished vs. malnourished hospitalised older subjects. In conclusion, the authors recommend using both NRS-2002 and SGA to detect malnutrition or risk of malnutrition in routine clinical practice of the geriatric department ward [8].

The last original article emphasises the role of albumin as a useful marker of in-hospital malnutrition and frailty. The prevalence of preexisting hypoalbuminemia at the time of discharge from the hospital was investigated using a sample of 9428 patients. Analysis of albumin levels at admission and at discharge was conducted by classes of albuminemia and then stratified by age. At the time of admission, hypoalbuminemia was found to be present in more than half of the sample, with no sex differences. The serum albumin level tended to decrease with age. The condition of marked and mild hypoalbuminemia was more prevalent in patients over 65 years of age. The authors conclude that albumin levels should be integrated into the routine assessment of patients, especially when dealing with nutritionally fragile populations [9].

In conclusion, the present Special Issue presents several aspects of assessment of nutritional status and prevention and treatment of nutritional deficiencies in different populations of older adults. Undoubtedly, future research will deepen our knowledge on this crucial public health issue.
